# Image dataset: UAV images and ground data of one ‘Bingo’ mandarin and two ‘Valencia’ orange rootstock trials conducted in Florida

**DOI:** 10.1016/j.dib.2024.111206

**Published:** 2024-12-05

**Authors:** Randall P. Niedz, Kim D. Bowman

**Affiliations:** Agricultural Research Service, U.S. Horticultural Research Laboratory, 2001 South Rock Road, Ft. Pierce, FL, USA

**Keywords:** Aerial imagery, Citrus, Plant breeding, Rootstock breeding, Flatwoods

## Abstract

The data are aerial images and ground tree measurement data of 3 citrus rootstock trials. Developing new citrus rootstock varieties requires field trials to test to identify selections with improved horticultural performance. A bud from a scion variety is grafted onto the rootstock and grown in a nursery until the grafted plant is ready to be planted in the field, which is in about one year. Trees in the field are assessed each year by measuring height, canopy diameter in 2 dimensions, overall health, and fruit number and quality factors when the trees begin to have a significant crop (∼3 years). Data collection of each tree is done manually. The image and ground data sets are of 3 rootstock trials that includes a 3-year-old Bingo mandarin hybrid trial of 206 trees, a 6-year-old Valencia orange trial of 643 trees, and a 7-year-old Valencia orange trials of 648 trees. Data for each trial includes aerial images and ground data of height, canopy diameters, and an overall health rating. The combination of ground validated measures and aerial images make this data set useful for building AI-based aerial image data collection applications. The data will be useful for 1) visualizing the effects of different rootstock selections and varieties on scion growth, effects that may not be fully captured with single measure metrics; and 2) development of image analysis applications and segmentation algorithms that can extract data from the images that are suitable for replacing some or all the ground measures.

Specifications Table

**Table:** 

Subject	Horticulture
Specific subject area	Aerial imagery to compliment and/or replace ground measures for citrus rootstock breeding trials.
Type of data	Image, table, raw.
Data collection	The images were captured with a DJI Phantom 4 Pro drone equipped with a 20 MP camera with a 1″ CMOS sensor. Images were standard red-green-blue (sRGB) and had a size 3:2 format of 5472 × 3648 pixels. The drone was flown at an altitude of 46 m, a speed of 12 kph, and flight lines with 80 % side-overlap and 80 % forward-overlap. Ground height and canopy width data was taken manually using extendable measuring sticks.
Data source location	Data collected at the USDA Research Farm (27.43717176349016, −80.42743420070789). Data stored by the United States, USDA National Agricultural Library (39.022807354728855, −76.92179156871161).
Data accessibility	Repository name: USDA Ag Data Commons [1] Direct URL to Rows 1–4 Bingo rootstock data: 10.15482/USDA.ADC/26946823 [2] Direct URL to Rows 5–16 and 17–28 Valencia rootstock data: 10.15482/USDA.ADC/26946841
Related research article	For the Valencia Rows 5–16 rootstock trial – [3] Bowman K.D., McCollum G., Seymour D.K. Genetic modulation of Valencia sweet orange field performance by 50 rootstocks under huanglongbing-endemic conditions. Frontiers in Plant Science, 14, art. no. 1,061,663 (2023). 10.3389/fpls.2023.1061663

## 1 Value of the Data

• *Unique perspective.* The images show what the trees looked like from above, a perspective not captured by ground imagery and measures. This perspective would be particularly useful for plant breeders and horticulturists studying rootstock selection trials as certain patterns and details may be easier to detect from images, which would be particularly true for images taken through time.
• Photo documentation. Images are effective at communicating complex information to a broader audience, including nonspecialists, compared to other measures and are useful for presentations and publications.
• *Quality data control.* Images useful to help ensure that other data are accurately recorded.
• *Image analysis applications.* The images in combination with the ground data can be used to develop image segmentation and analysis applications to extract data from the images that are suitable for complementing and/or replacing some or all the ground measures.

## 2 Background

Developing new and improved citrus varieties requires that selected individuals be field tested to evaluate the performance of the different genotypes under commercial conditions. Performance includes measuring multiple traits of importance and includes fruit yield and tolerance to abiotic stresses such as flooding from heavy rainfall, drought during dry spells, salinity in the irrigation water from saltwater intrusion near coastal areas, freeze damage, and inadequate nutrition from poor nutrient uptake. Some biotic stresses include the Diaprepes root weevil, bacterial canker, blackspot, Citrus tristeza virus, root rot fungi such as Phytophthora, and citrus greening disease (Huanglongbing) as the most serious citrus disease. A citrus tree is grown as a composite tree that is composed of a scion variety grafted onto a rootstock variety. The rootstock is selected because of its tolerance to various stresses and its positive effects on fruit production, including reducing the juvenility phase for earlier fruit production. A rootstock trial to select individuals for further testing will include multiple rootstock selections and a single commercial scion variety. Also, the trial will include some standard commercial rootstocks for comparison. Tree growth and fruit data is collected once a year over the timespan of the trial, which is at least 8–10 years. Data is collected on every tree in the trial and a typical trial will include multiple acres and hundreds of trees. Data collection is a laborious process and therefore expensive. The motivation for collecting aerial imagery was that quicker and cheaper methods for collecting tree performance and fruit data would be useful for citrus breeding.

## 3 Data Description

The data are of three rootstock trials documented as images and ground data. The ground data is canopy diameter in 2 dimensions and canopy height (both measured in centimeters), and a subjective score of canopy health on a 0–5 scale (0 = dead; 5 = excellent canopy thickness and leaf color). An Excel spreadsheet for each trial includes ten column fields (Table 1). The datasets are as follows –

**Fig. 1:**
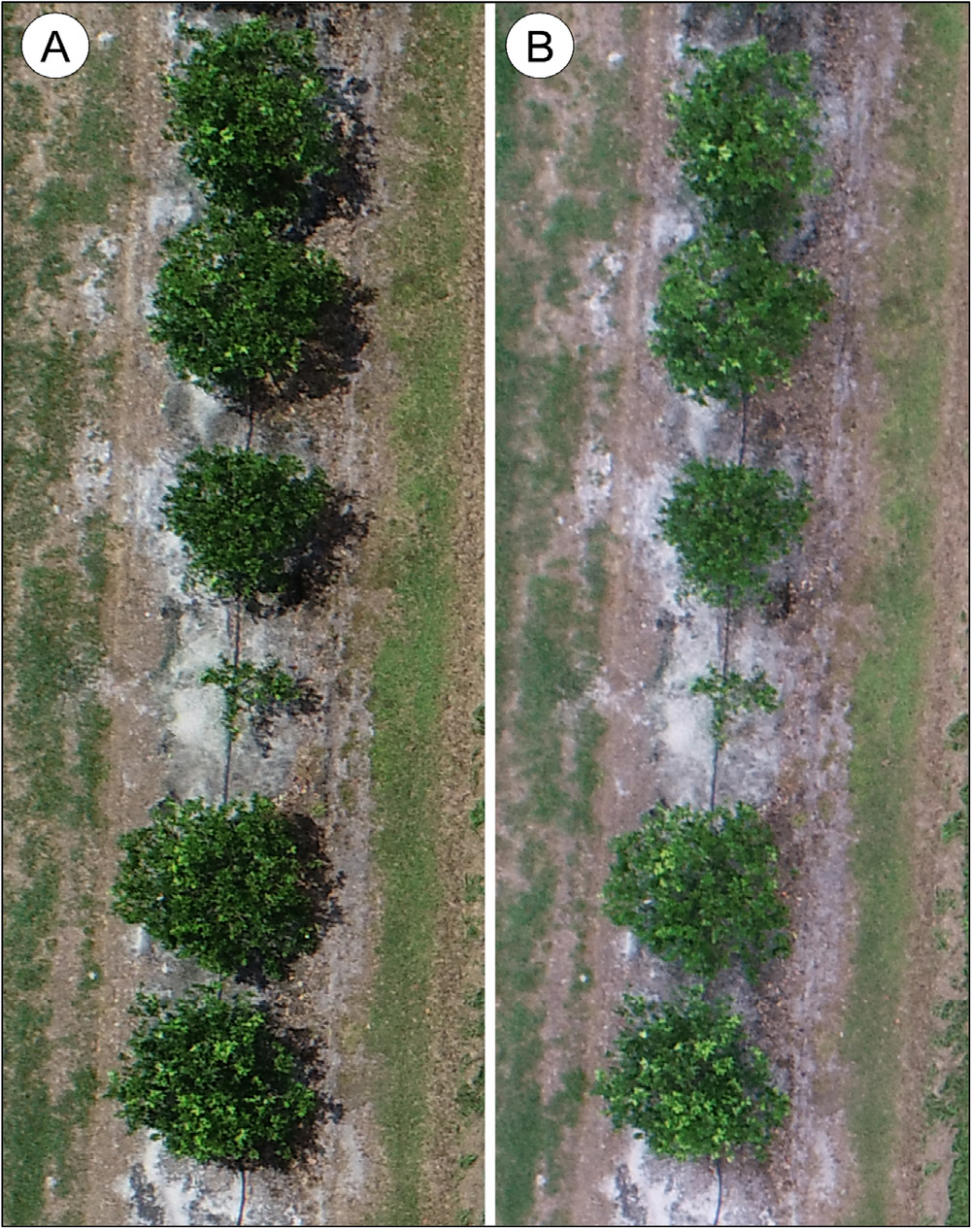
Image of trees under sunny vs overcast conditions. A) Trees under sunny conditions from image **DJI_0054_R5-R28_Valencia_sunny.jpg**. B) Trees under overcast conditions from image **DJI_0203_R5-R28_Valencia_overcast.jpg**.

1. Bingo mandarin hybrid rootstock trial. The dataset is included in one folder that contains 48 files – 46 image files, 1 Excel spreadsheet, and 1 image with the rows and tree spaces labeled. The labeled image is a composite image of the 46 individual images and was created for labeling rows and tree space numbers.
2. Valencia 5–16 and 17–28 rootstock trials. The dataset is included in one folder that contains 304 files – 300 image files, 2 Excel spreadsheets, and 2 images with the rows and tree spaces labeled. The labeled images are composite images from the 300 images and was created for labeling rows and tree space numbers. The dataset includes aerial images from 2 flights taken the same day and flying the same pre-programmed flight program. Each flight was programmed to fly over and photograph rows 5–28, thereby capturing images of the 2 rootstock trials, 5–16 and 17–28. One flight, images labeled 27–176, was taken when it was partially sunny resulting in some images with shadows (Fig. 1A) and others with no shadows when the sun was behind a cloud. The other flight, images labeled 172–327, was taken when the sky was overcast resulting in uniform lighting and no shadows (Fig. 1B). Each image is labeled to indicate if it is part of the sunny or overcast sets. For example, image labeled **DJI_0033_R5-R28_Valencia_sunny.JPG** was taken from the partially sunny flight, and the image labeled **DJI_0183_R5-R28_Valencia_overcast.JPG** was taken from the overcast flight.

**Table 1:** Excel spreadsheet columns included for each rootstock trial.

Column labels	Description
ID	Each spreadsheet entry numbered.
Row #	Row number.
Tree space #	Space number in the row. Trees are assigned to a space.
Scion	Scion variety.
Plant date	The date that the trees were planted.
Rootstock	Rootstock variety or selection.
Canopy height, [date measured]	Height of the tree (cm). Date measured.
Canopy width N-S, [date measured]	N-S width of the tree (cm). The down-the-row width. Date measured.
Canopy width E-W, [date measured]	E-W width of the tree (cm). The across-the-bed width. Date measured.
Canopy Health, [date measured]	Visual overall health rating. Rating scale 0–5 where 0 = dead, and 5 = fully healthy. Date measured.

## 4 Experimental Design, Materials and Methods

**Fig. 2:**
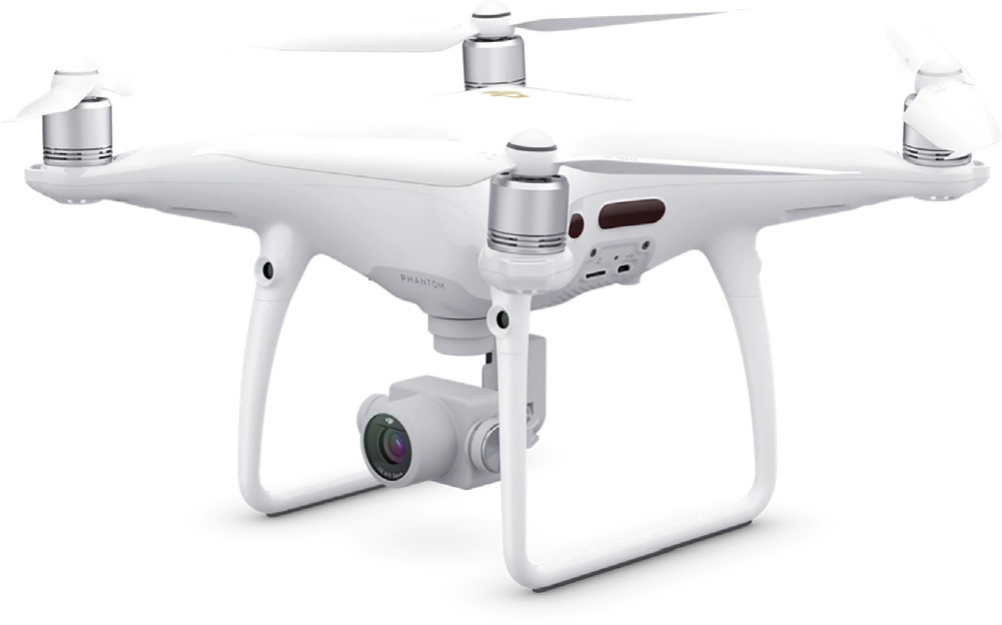
DJI Phantom 4 Pro UAV quadcopter used for image acquisition.

**Fig. 3:**
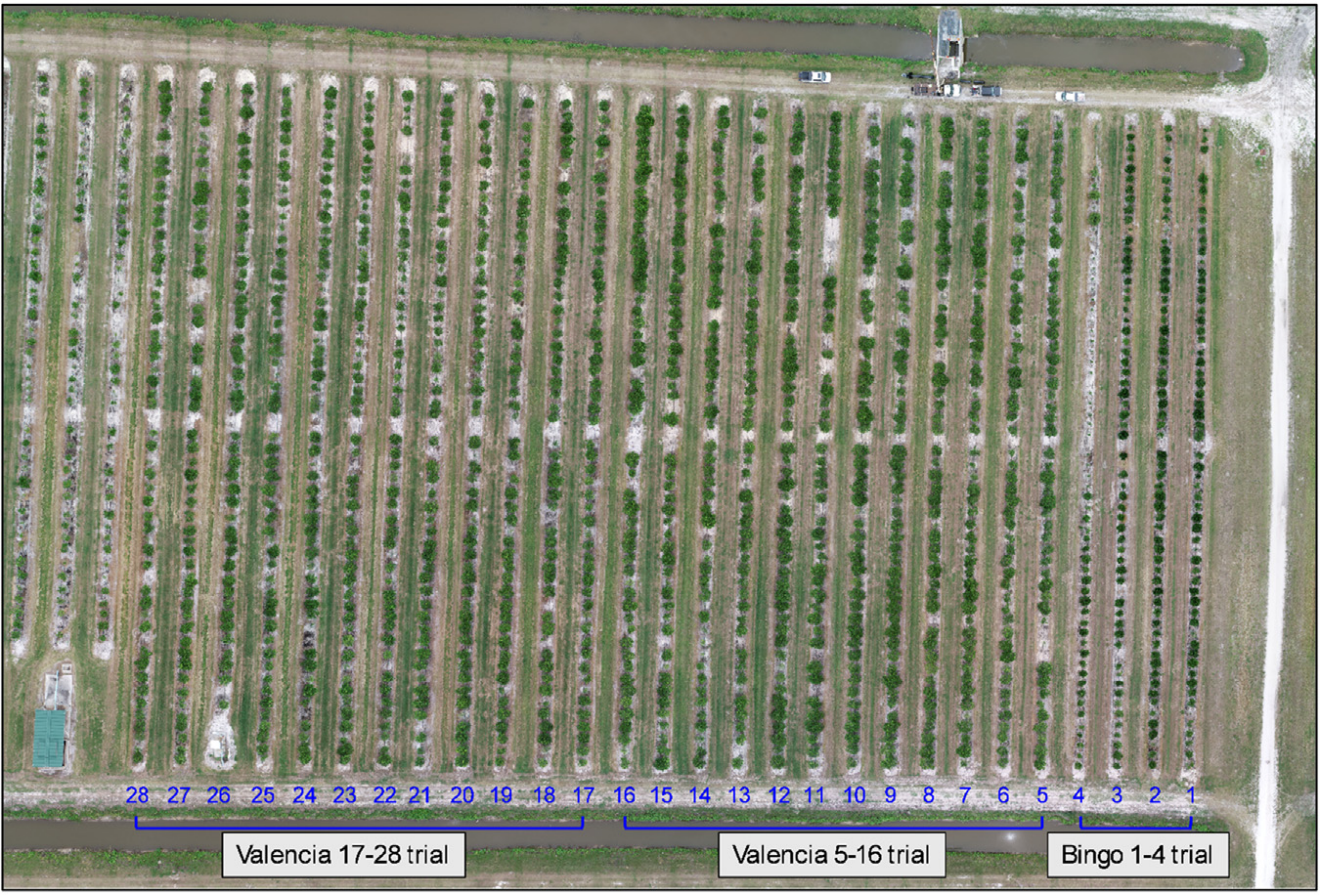
Aerial composite image of the 3 rootstock trials field plot. Rows are oriented south (bottom) to north (top). Row numbers in blue. Bingo rootstock trial located in rows 1–4. Valencia 5–16 rootstock trial located in rows 5–16. Valencia 17–28 rootstock trial located in rows 17–28. Multiple rootstocks tested in each trial.

The images were captured with a DJI Phantom 4 Pro (DJI, Shenzhen, China) (Fig. 2) drone equipped with a 20 MP camera with a 1″ CMOS sensor. Images were standard red-green-blue (sRGB), had a size 3:2 format of 5472 × 3648 pixels, and were JPG format. The drone was flown at an altitude of 46 m, a speed of 12 kph, and flight lines with 80 % side-overlap and 70 % forward-overlap. Ground height and canopy width data were taken manually using extendable measuring sticks. Rows 5–28 containing the 2 Valencia rootstock trials were flown twice the same day – once when the sky was partially cloudy and once when the sky was overcast. The images of both flights, labeled sunny and cloudy, are included. The 3 rootstock trials were located at the USDA research farm site (Fig. 3) and were as follows:

1. Bingo mandarin hybrid rootstock trial (Fig. 4) planted in 2018. Located in rows 1–4 and included 14 rootstock varieties and selections.

**Fig. 4:**
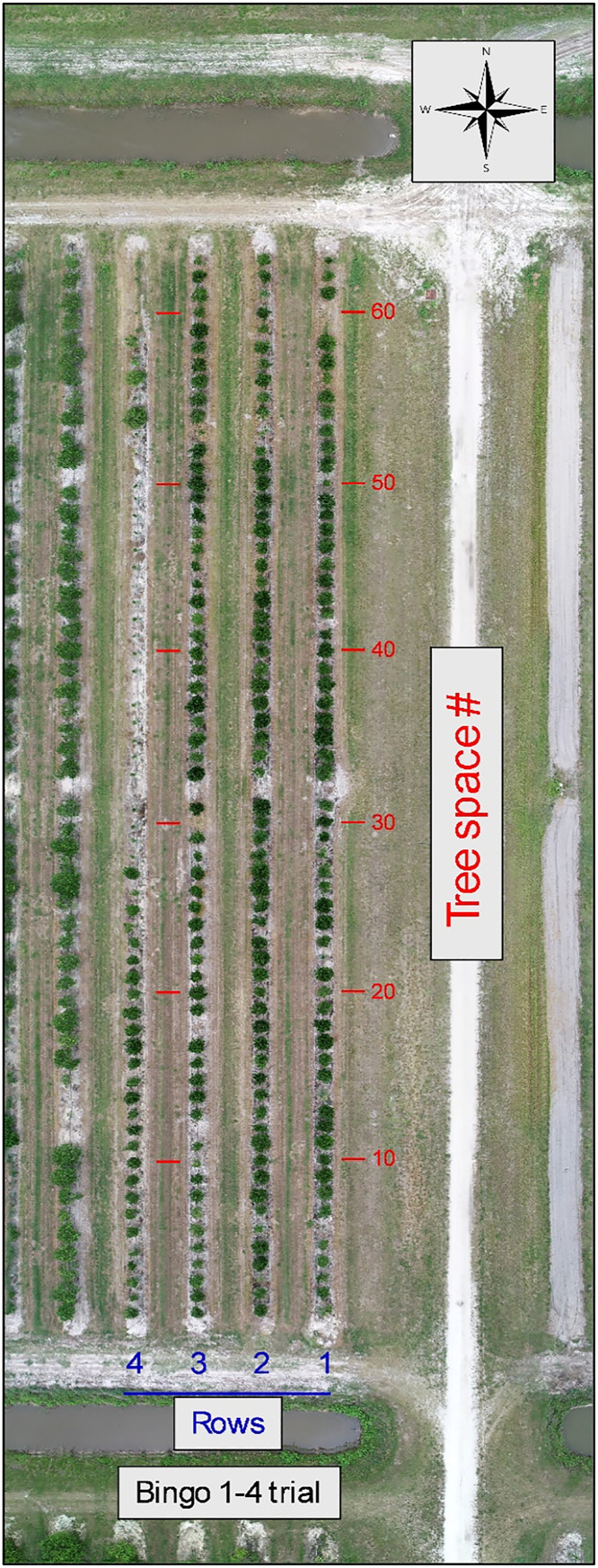
Aerial composite image of the Bingo mandarin hybrid rootstock trial. Row (blue) and tree space numbers (red) added for orientation.
2. Valencia rootstock trial rows 5–16 (Fig. 5) planted 2014. Located in rows 5–16 and included 67 rootstock varieties and selections.

**Fig. 5:**
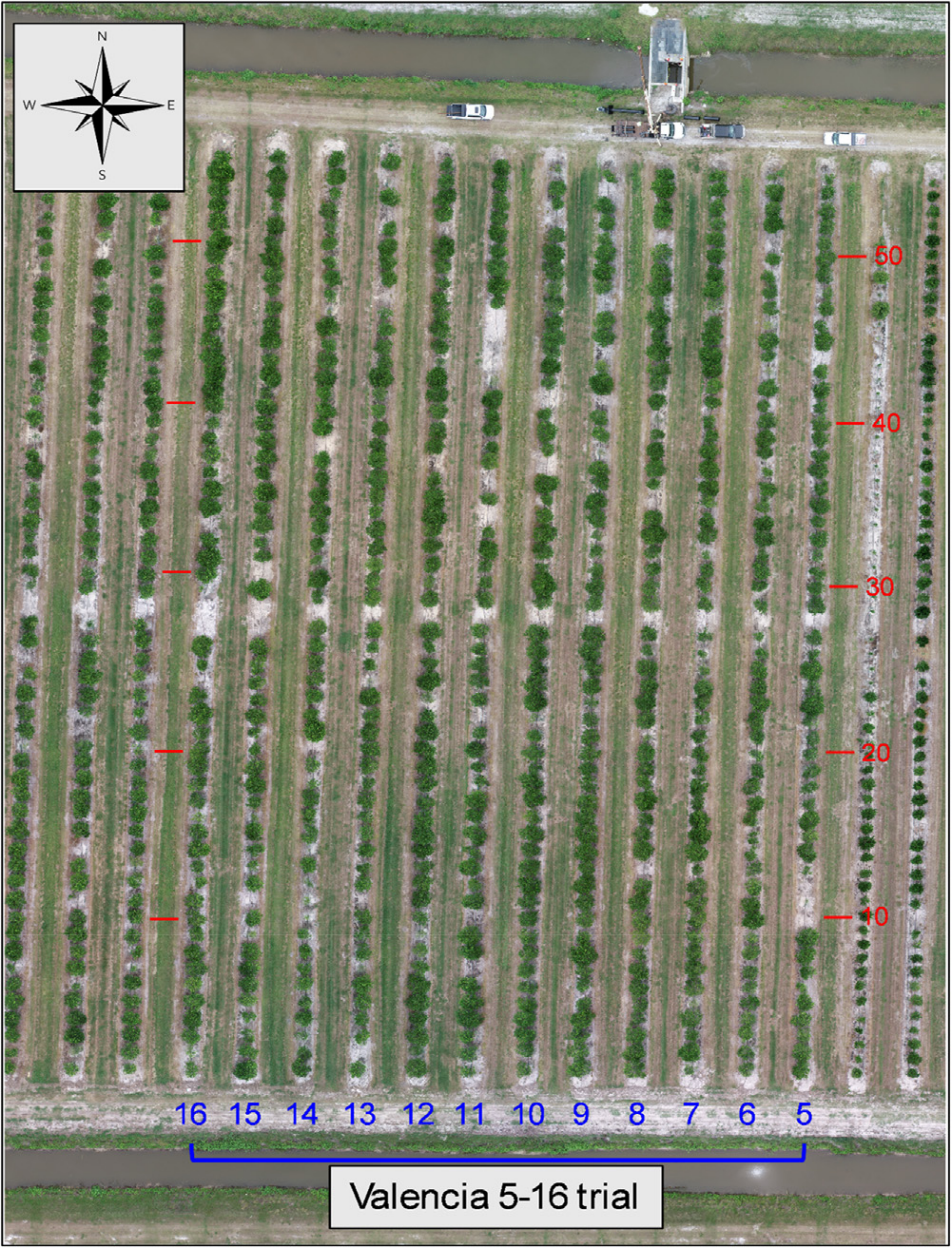
Aerial composite image of the Valencia 5–16 rootstock trial. Row (blue) and tree space numbers (red) added for orientation.
3. Valencia rootstock trial rows 17–28 (Fig. 6) planted 2015. Located in rows 17–28 and included 69 rootstock varieties and selections.

**Fig. 6:**
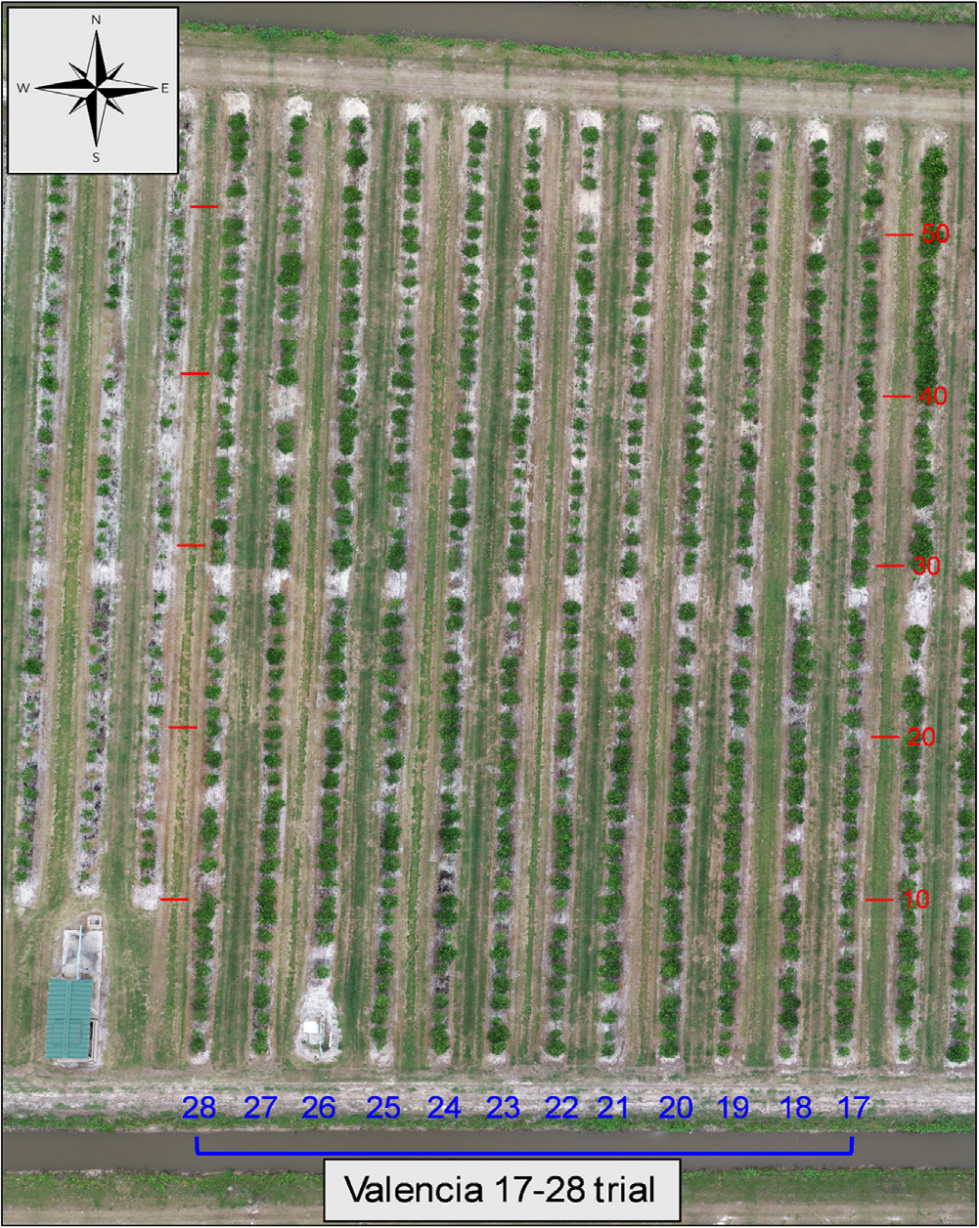
Aerial composite image of the Valencia 17–28 rootstock trial. Row (blue) and tree space numbers (red) added for orientation.

## Limitations

One limitation of the data sets is that they are for one year. A citrus rootstock field trial typically requires at least 8–10 years, 3 years before the trees flower and set fruit, and another 5–7 years to assess fruit production. Ground data is taken every year of the trial and fruit data every year that the trees produce fruit. Ideally, aerial images would be taken each year of the trial. Another limitation is that the aerial images are a top-down view and taken at a single altitude of 46 m. Other types of aerial images such as oblique views and lower altitudes for more detail might could also be useful. The aerial images are JPG format. JPG uses lossy compression, which means some image data is discarded to reduce file size, and because the data is compressed artefacts can be introduced. RAW format would provide much more image information including a wider dynamic range and color spectrum.

## Ethics Statement

1. The authors have read and followed the ethical requirements for publication in Data in Brief and confirm that the current work does not involve human subjects, animal experiments, or any data collected from social media platforms.
2. The images of this work have not been published previously.
3. The manuscript is not under consideration for publication elsewhere.
4. Each author has approved the manuscript for publication, along with the responsible authorities where the work was carried out, either tacitly or explicitly.
5. If accepted, the article will not be published elsewhere in the same form, whether itʼs in English, another language, or electronically without the copyright-holder's consent.

## CRediT Author Statement

**Randall Niedz:** Conceptualization, Methodology, Writing- Original draft preparation, Writing- Reviewing and Editing, Image Data Curation. **Kim Bowman:** Methodology, Writing- Reviewing and Editing, Ground Data Curation.

## Data Availability

Ag Data CommonsUAV image and ground data of citrus ‘Bingo’ mandarin hybrid (Citrus reticulata, Blanco) rootstock trial. (Original data). Ag Data CommonsUAV image and ground data of two citrus ‘Valencia’ orange (Citrus sinensis [L.] Osbeck) rootstock trials. (Original data).
